# Serum anti-PLA2R antibody and glomerular PLA2R deposition in Chinese patients with membranous nephropathy: A cross-sectional study

**DOI:** 10.1097/MD.0000000000007218

**Published:** 2017-06-16

**Authors:** Lu Pang, Ai-Min Zhang, Hai-Xia Li, Jia-Lin Du, Li-Li Jiao, Nan Duan, Yi Liu, Dan Yu

**Affiliations:** a Department of Clinical Laboratory, Peking University First Hospital, Beijing; b Department of Clinical Laboratory, Tianjin Nankai Hospital, Tianjin, The People's Republic of China.

**Keywords:** glomerular PLA2R deposition, membranous nephropathy, serum anti-PLA2R antibody

## Abstract

M-type phospholipase A2 receptor (PLA2R) is the major target antigen in primary membranous nephropathy (PMN). Previous studies have evaluated the diagnostic value of serum anti-PLA2R antibody. However, the correlation of serum anti-PLA2R antibody and glomerular PLA2R deposition, and their association with clinical characteristics need to be further evaluated.

A total of 136 patients were involved as inception group because serum anti-PLA2R antibody and glomerular PLA2R antigen were simultaneously measured. We examined serum anti-PLA2R antibody by ELISA and glomerular PLA2R deposition by immunofluorescence assay.

Positive serum anti-PLA2R antibody and glomerular PLA2R deposition were seen in 58.8% (80/136) and 95.6% (130/136) patients, respectively (*P* < .001). Proteinuria, serum total protein, serum albumin, serum creatinine, and estimated glomerular filtration rate (eGFR) had significant differences between patients with serum anti-PLA2R antibody and those without. Serum anti-PLA2R antibody levels were correlated with serum albumin, serum creatinine, eGFR, and proteinuria. Glomerular PLA2R deposition intensities were weakly correlated with proteinuria. Unexpectedly, there was a positive correlation rather than a negative correlation between glomerular PLA2R deposition intensity and eGFR.

In conclusion, serum anti-PLA2R antibody is more closely correlated with disease activity and renal function than glomerular PLA2R deposition.

## Abstract 2

Supplemental Digital Content is available in the text

## 1 Introduction

Membranous nephropathy, a major cause of adults nephrotic syndrome,^[1]^ is classified into primary membranous nephropathy (PMN) and secondary membranous nephropathy related to various conditions, including autoimmune disease, infections, cancer, and drug intoxication.^[2]^

The discovery of autoantibody against M-type phospholipase A2 receptor (PLA2R), a 185 kDa type I transmembrane glycoprotein expressed on glomerular podocytes, was an important milestone in understanding of PMN.^[3]^ A genome-wide association study elaborated the genetic evidence that PLA2R involved in the development of PMN.^[4]^ Circulating anti-PLA2R antibodies could be detected in the majority of patients with PMN, while could not be detected in patients with other glomerular diseases and thus serum anti-PLA2R antibody was suggested to be a promising marker for the differential diagnosis of PMN.^[5]^

Patients with positive serum anti-PLA2R antibody often have positive glomerular PLA2R deposition.^[6]^ Although the diagnostic significance of serum anti-PLA2R antibody has been confirmed by several groups, the relevance of serum anti-PLA2R antibody with glomerular PLA2R deposition, and the relationship between PLA2R-related biomarkers and other biomarkers have been addressed in just few studies. Thus, we performed our retrospective study to compare the value of serum anti-PLA2R antibody and glomerular PLA2R deposition in reflecting disease activity and renal function.

## 2 Patients and methods

### 2.1 Patients and samples

**Figure 1:**
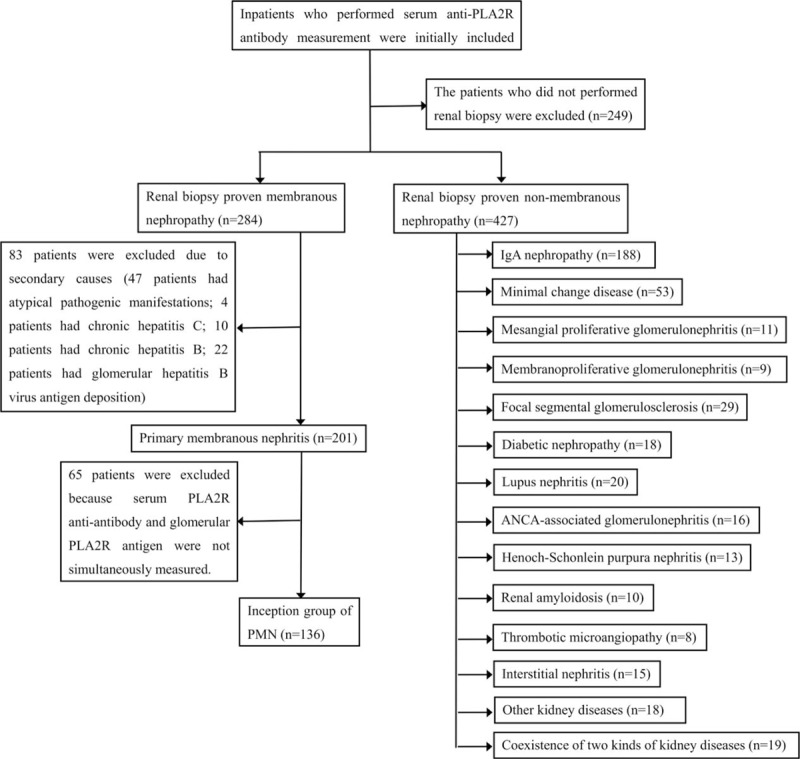
Schematic illustration of patient recruitment. The detail information of the 19 patients who had 2 kinds of kidney diseases are shown in Supplemental Table.

In total, 960 inpatients who performed serum anti-PLA2R antibody measurement between August 2015 and December 2016 were initially reviewed retrospectively from Peking University First Hospital. The patients who did not performed renal biopsy were excluded. Thus, 284 patients with renal biopsy proven MN and 427 patients with biopsy proven non-MN were included. Of all the MN patients, 83 patients were clinically ruled out for secondary MN, including systemic autoimmunity diseases, viral hepatitis B and C and HIV infection, neoplastic conditions, and exposure to toxic agents. Hence, 136 patients were selected as inception group because serum anti-PLA2R antibody and glomerular PLA2R antigen were simultaneously measured (Fig. 1). This study was approved by the ethics committee of Peking University First Hospital.

### 2.2 Therapeutic interventions

A total of 25% (34/136) patients received immunosuppressive treatment at the time of measurement of serum anti-PLA2R antibody and glomerular PLA2R deposition. Immunosuppressive interventions included glucocorticoids (20.59%, 28/136), cyclophosphamide (7.35%, 10/136), tripterygium wilfordii (5.88%, 8/136), cyclosporin A (5.15%, 7/136), leflunomide (1.74%, 2/136), mycophenolate mofetil (0.74%, 1/136), and tacrolimus (0.74%, 1/136).

### 2.3 Measurement of circulating anti-PLA2R antibody, glomerular PLA2R deposition, and glomerular IgG4 deposition

Serum anti-PLA2R antibody was measured by ELISA test using previously validated commercial kits (EUROIMMUN, lübeck, Germany) according to the manufacturer's instructions as previously reported.^[7]^ The results were considered as negative for <20 relative units (RU)/mL and positive for ≥20 RU/mL according to the manufacturer's protocol.

**Figure 2:**
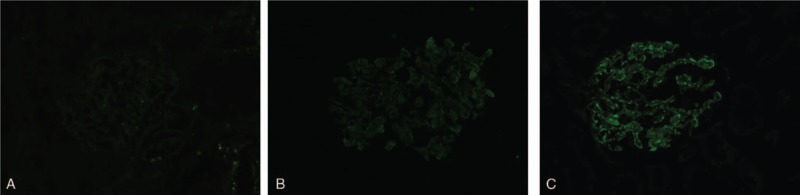
Immunofluorescence staining of M-type phospholipase A2 receptor (PLA2R) in glomerular deposition (×400). Semiquantitative glomerular PLA2R deposition intensities were evaluated by pathologists and scored as + (weak, A), ++ (moderate, B), or +++ (strong, C).

PLA2R and IgG4 in glomerular deposition were measured by immunofluorescence assay according to the method described previously.^[8,6]^ Semiquantitative glomerular PLA2R deposition intensities were evaluated by pathologists and scored as + (weak), ++ (moderate), or +++ (strong) (Fig. 2). Qualitative glomerular IgG4 deposition was evaluated by pathologists. Positivity of glomerular IgG4 expression was defined as fine granular pattern staining along the glomerular capillary walls.

### 2.4 Measurement of proteinuria and serum biomarkers

Urinary total protein (Pyrogallol red-molybdate method) was measured by 7180 automatic biochemical analyzer (Hitachi). For proteinuria, there was 1 missing value in patient with negative serum anti-PLA2R antibody and positive glomerular PLA2R deposition. Serum total protein (Biuret method), serum albumin (Bromcresol green method), serum creatinine (Jaffe method), and serum urea (Urease methods) were measured by Synchron DXC800 automatic biochemical analyzer (Beckman Coulter). Estimated glomerular filtration rate (eGFR) levels were calculated using the Chronic Kidney Disease Epidemiology Collaboration equation recommended by the Kidney Disease Improving Global Outcomes.^[9]^

### 2.5 Statistical analysis

All analyses were performed using the SPSS software version 19 for Windows (IBM, Chicago, IL). Data are presented as median (interquartile range). The case with missing value was deleted. Mann–Whitney *U* test was used to compare differences between continuous data. Chi-square (χ^2^) test was used to compare differences between categorical data. The correlation between serum anti-PLA2R antibody and other clinical biomarkers were analyzed by Spearman coefficient. A two-tailed *P* value < .05 was considered statistically significant.

## 3 Results

### 3.1 Distribution of serum anti-PLA2R antibody and glomerular PLA2R deposition of inception group

**Figure 3:**
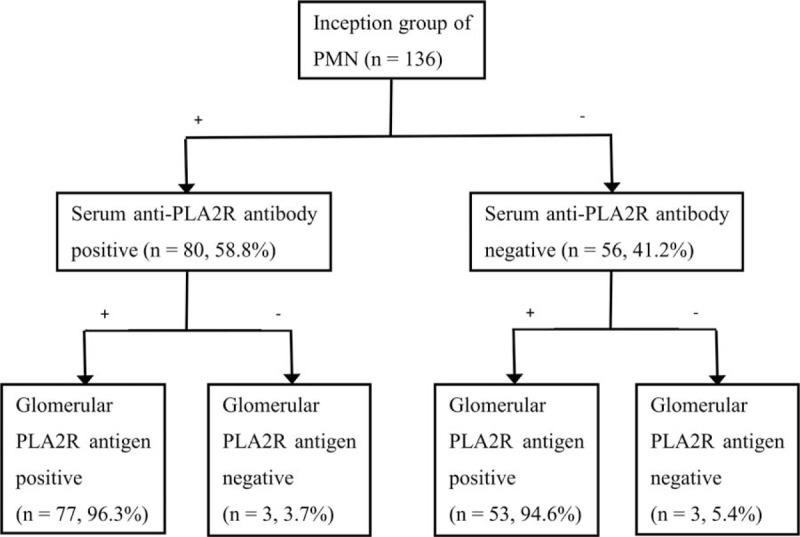
Distribution of serum anti-M-type phospholipase A2 receptor (PLA2R) antibody and glomerular PLA2R deposition of inception group. −, negative; +, positive.

As shown in Table 1 and Fig. 3, 58.8% patients (80/136) had positive serum anti-PLA2R antibody, while 41.2% patients (56/136) were negative. Serum anti-PLA2R antibody levels showed a wide range of values in our study from negative result to over 1500.00 RU/mL (beyond the detection limit). The median level of serum anti-PLA2R antibody was 93.00 RU/mL. (Interquartile range, 23.63–162.38 RU/mL) (Supplemental Figure). The patients (n = 427) who had renal biopsy proven nonmembranous nephropathy were negative for serum anti-PLA2R antibody. In our study, the specificity of serum anti-PLA2R antibody for PMN is 100% and the sensitivity is 58.8%. As for the glomerular PLA2R deposition of inception group, 95.6% patients (130/136) were positive and 4.4% patients (6/136) were negative (Table 1).

**Table 1:**
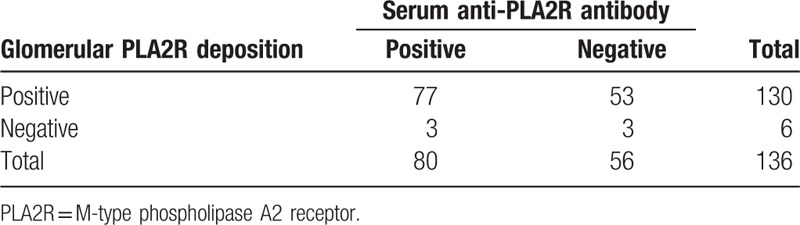
Serum anti-PLA2R antibody and glomerular PLA2R deposition of inception group.

**Figure 4:**
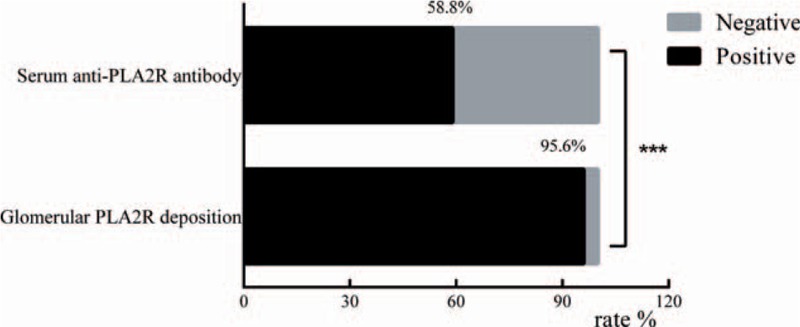
Positive rates of serum anti-M-type phospholipase A2 receptor (PLA2R) antibody and glomerular PLA2R deposition of inception group. Chi-square test was used to compare the positive rates. ^∗∗∗^*P* < .001.

Positive rates of serum anti-PLA2R antibody and glomerular PLA2R deposition were 58.8% and 95.6%, respectively (*P* < .001) (Fig. 4). Literature review revealed that the positive rates of serum anti-PLA2R antibody and glomerular PLA2R deposition were incongruent, varying from 52% to 82% and 69% to 92% across current studies (Tables 2 and 3).

**Table 2:**
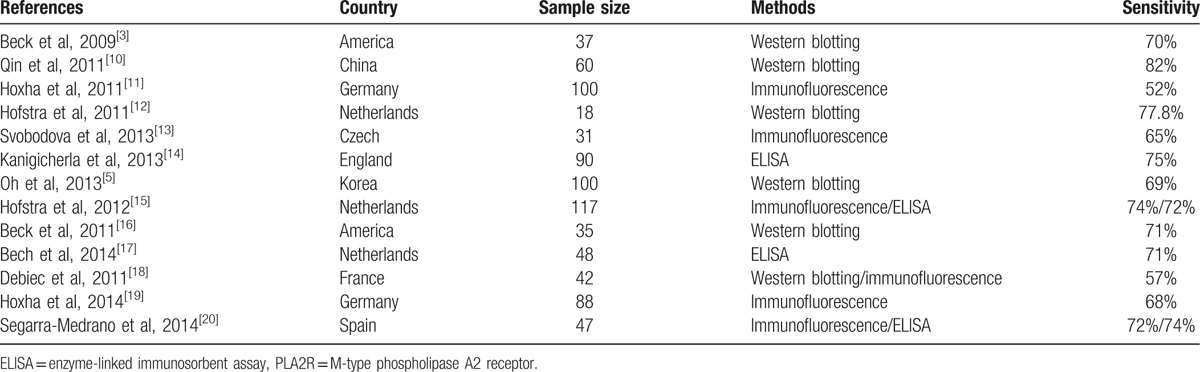
The sensitivity of serum anti-PLA2R antibody in previous studies.

**Table 3:**
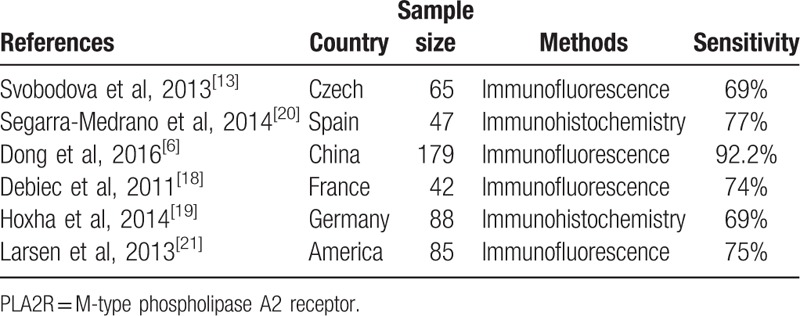
The sensitivity of tissue deposition of PLA2R in previous studies.

### 3.2 The relationship between serum anti-PLA2R antibody and glomerular PLA2R deposition

Of 80 patients who had positive serum anti-PLA2R antibody, 77 had positive glomerular PLA2R deposition (96.3%) and 3 were negative. Of 56 patients who were negative for serum anti-PLA2R antibody, 53 (94.6%) had positive glomerular PLA2R deposition and 3 were negative (Table 1 and Fig. 3). The overall agreement was 58.8% and Kappa value was 0.019.

**Figure 5:**
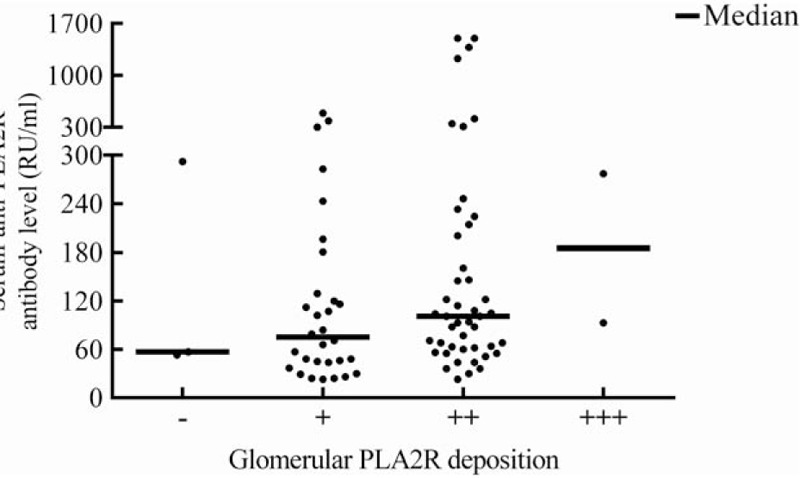
Correlation between serum anti-M-type phospholipase A2 receptor (PLA2R) antibody (RU/mL) and glomerular PLA2R deposition (0–3+ strength of staining). Spearman correlation coefficient = 0.18, *P* = .11. Continuous lines indicate median concentrations. The medians of serum anti-PLA2R antibody were 57 (−), 75 (+), 101 (++), and 185 (+++) RU/mL.

We also analyzed the correlation between serum anti-PLA2R antibody and glomerular PLA2R deposition in serum PLA2R antibody positive patients of inception group. Although the Spearman correlation coefficient is 0.18 (*P* = .11), the high level of serum PLA2R antibody was related to the strong deposition of glomerular PLA2R antigen (Fig. 5).

### 3.3 The relationship between PLA2R and IgG4 in glomerular deposition

Of 136 patients with PMN, 128 (94.1%) patients simultaneously had PLA2R and IgG4 in glomerular deposition, 5 patients had IgG4 but no PLA2R deposition, and 2 patients had PLA2R but no IgG4 deposition. Both PLA2R and IgG4 were negative in 1 patient.

### 3.4 The relationship between PLA2R-related biomarkers and other clinical biomarkers

We observed the differences of clinical parameters between serum anti-PLA2R antibody-positive and -negative patients of inception group. There were significant differences in proteinuria, serum total protein, serum albumin, serum creatinine, and eGFR, but no significant differences in serum urea (Table 4). We also compared the differences between serum anti-PLA2R antibody-positive (n = 77) and -negative (n = 53) patients in the premise of positive glomerular PLA2R deposition. There were significant differences in proteinuria, serum total protein, serum albumin, serum creatinine, and eGFR between 2 groups (Table 5).

**Table 4:**
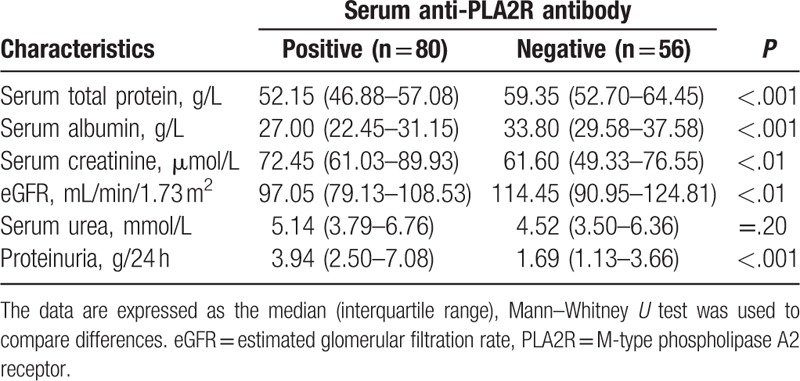
The differences of clinical parameters between positive and negative patients both of serum anti-PLA2R antibody.

**Table 5:**
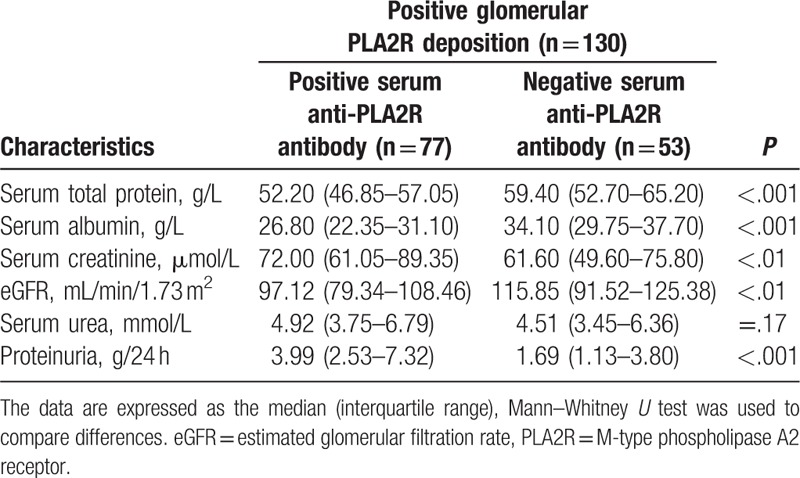
The differences between serum anti-PLA2R antibody-positive (n = 77) and -negative (n = 53) patients in the premise of positive glomerular PLA2R deposition.

We analyzed the correlation between PLA2R-related biomarkers and other clinical biomarkers in serum anti-PLA2R antibody positive patients and glomerular PLA2R deposition positive patients. Serum anti-PLA2R antibody levels were correlated with serum albumin, serum creatinine, eGFR, and proteinuria but not correlated with serum urea and serum total protein. Glomerular PLA2R deposition intensities were weakly correlated with serum total protein, serum albumin, and proteinuria. Unexpectedly, there was a positive correlation between glomerular PLA2R deposition intensity and eGFR (Table 6).

**Table 6:**
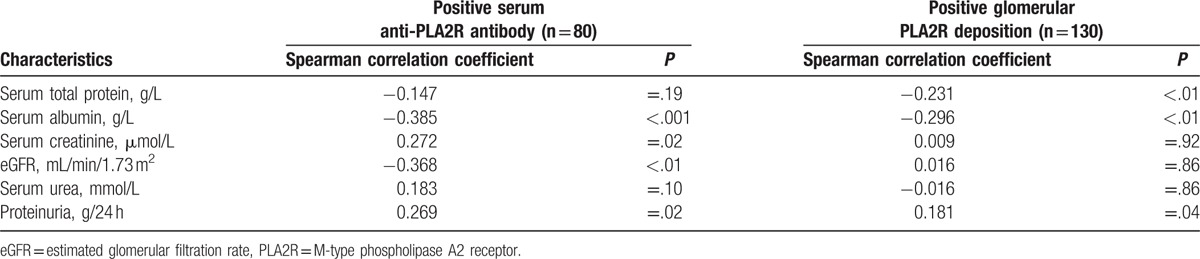
The correlation between PLA2R-related biomarkers and other clinical biomarkers in positive serum anti-PLA2R antibody patients and positive glomerular PLA2R deposition patients.

## 4 Discussion

PMN is the most common pathological form of nephrotic syndrome in adults. In our study, we compared the clinical values of serum anti-PLA2R antibody and glomerular PLA2R deposition in a Chinese cohort. Although the diagnostic significance of serum anti-PLA2R antibody has been confirmed by several groups, the relevance of serum anti-PLA2R antibody with glomerular PLA2R deposition, and the relationship between PLA2R-related biomarkers and other biomarkers have been addressed in just few studies.

A series of previous studies were incongruent in the diagnostic value of serum anti-PLA2R antibody, especially in sensitivity, varying from 52% to 82% across current studies. Two recent meta analyses assessed the overall diagnostic values of serum anti-PLA2R antibody in PMN detection, demonstrating the overall sensitivity to be 74% and 78% and the overall specificity to be 95% and 99%.^[22]^ PLA2R antigen in glomerular deposition was found in 69% to 92% PMN patients. Our data indicated 58.8% patients with PMN had circulating anti-PLA2R antibody and 95.6% patients had positive glomerular PLA2R staining. Some researchers had a suspicion that the proportion of PLA2R-related PMN cases in Chinese patients with PMN was higher than other countries.^[6]^ Our study displayed that the higher sensitivity of glomerular PLA2R deposition was seen in Chinese cohort, which was consistent with the discovery from Dong et al,^[6]^ but the sensitivity of serum anti-PLA2R antibody of Chinese patients with PMN was not apparently higher than those of other countries. Multicenter clinical studies will be definitely needed to clarify whether Chinese patients with PMN have higher proportion of PLA2R-related cases.

**Figure 6:**
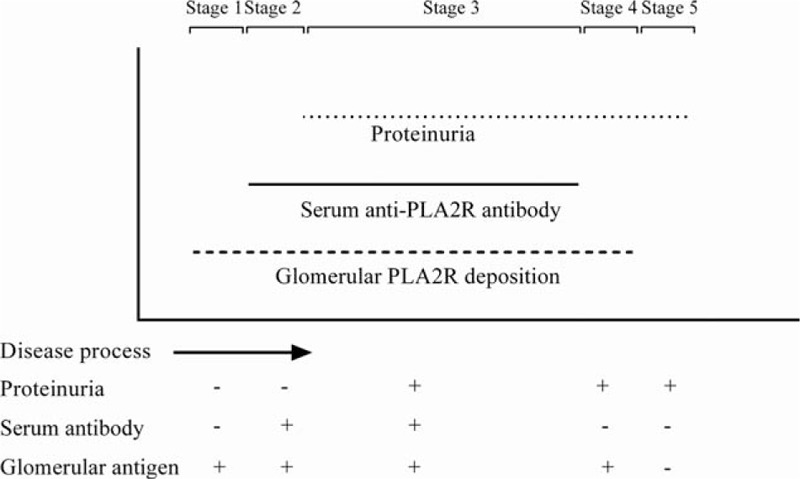
The temporal sequence of serum anti-PLA2R antibody, glomerular PLA2R deposition, and proteinuria in the process of PMN progression. −, negative; +, positive. PLA2R = M-type phospholipase A2 receptor, PMN = primary membranous nephropathy.

As shown in Fig. 6, Francis et al^[23]^ have clarified glomerular PLA2R deposition may precede the appearance of circulating anti-PLA2R antibody (stage 1), and at some point (the end of stage 2), the subepithelial PLA2R deposition will cause sufficient podocytes injury to lead to detectable proteinuria. When serum anti-PLA2R antibody disappear, glomerular PLA2R deposition will start to attenuate, which means glomerular PLA2R deposition will continue to be detected after the serum anti-PLA2R antibody disappears (stage 4). As previously reported in the French cohort^[18]^ and Czech cohort,^[13]^ we found that 53 patients in our study had no circulating anti-PLA2R antibody although they had positive glomerular PLA2R deposition. These discordant finding might be explained by the stage 1, in which the appearance of glomerular PLA2R deposition precede serum anti-PLA2R antibody, and stage 4, in which the clearance of serum anti-PLA2R antibody precede glomerular PLA2R deposition. On the other hand, 3 patients had positive serum anti-PLA2R antibody but did not have detectable PLA2R in glomerular deposition. We suspect these autoantibodies were false positive.

In this study, among 136 patients, 5 patients had IgG4 but no PLA2R deposition. In this situation, a possible explanation for the result is that there could be another antigenic target of autoantibody driving the disease. For example, thrombospondin type-1 domain-containing 7A was recently described as a 2nd antigenic target in PMN.^[24]^ On the other hand, 2 patients had PLA2R but no IgG4 deposition. It is interesting that both have negative serum anti-PLA2R antibody, which means the patients may in the stage 1 or stage 4 of the disease process. As for the patient who had dual negative results of PLA2R and IgG4 deposition, one possibility is that the patient was misdiagnosed as PMN because of negative serum anti-PLA2R antibody and positive antiglomerular basement membrane antibody. However, we have also noticed the recently published article by Dong et al,^[6]^ in which 2.8% (5/179) patients had dual negative results of PLA2R and IgG4 deposition.

Several studies had clarified that serum anti-PLA2R antibody had high sensitivity and specificity but with several limitations, including inappropriate time points, insufficient numbers of patients, and different measurement methods. In order to compare the clinical significance of serum anti-PLA2R antibody and glomerular PLA2R deposition, we assessed the relationship between serum anti-PLA2R antibody and glomerular PLA2R deposition only in inception group, in which the serum samples were collected at the time of renal biopsy. In our study, we observed that the high level of serum anti-PLA2R antibody was related to the strong intensity of glomerular PLA2R deposition although the Spearman correlation coefficient was 0.18. Larger sample will be needed to clarify whether there is a positive correlation.

Previous studies have shown that serum anti-PLA2R antibody is a good marker for PMN diagnosis and disease activity monitoring. On the contrary, Hill et al^[8]^ revealed that serum anti-PLA2R antibody cannot reflect the severity of PMN. Whether anti-PLA2R antibody can reflect the severity of PMN is still controversial due to the different populations and methods for the determination of serum anti-PLA2R antibody. In our study, proteinuria, serum total protein, serum albumin, serum creatinine, and eGFR showed significantly differences between patients with serum anti-PLA2R antibody and those without. Moreover, serum anti-PLA2R antibody levels were correlated with serum albumin, serum creatinine, eGFR, and proteinuria. Although there is no statistical significance, the level of serum urea is higher in positive serum anti-PLA2R antibody group than negative group. Serum urea is a less robust marker than serum creatinine because the former is more susceptible to amino-acid administration. It is conceivable that there could be significant difference when more patients are recruited.

Glomerular deposition of PLA2R provides a supplementary marker in the diagnosis of PMN, and it can be detected in patients with PMN and without detectable serum anti-PLA2R antibody. However, due to the weak correlation, weak overall agreement, and low Kappa value between serum anti-PLA2R antibody and glomerular PLA2R deposition, we have a suspicion that whether glomerular PLA2R deposition intensity can reflect renal function. In this study, glomerular PLA2R deposition intensities were weakly correlated with proteinuria. Unexpectedly, there was a positive correlation rather than a negative correlation between glomerular PLA2R deposition intensity and eGFR. These results indicate that serum anti-PLA2R antibody is more closely correlated with disease activity and renal function than glomerular PLA2R deposition.

In order to avoid the confounding bias, we further compared the differences between serum anti-PLA2R antibody-positive and -negative patients in the premise of positive glomerular PLA2R deposition. There were significant differences in proteinuria, serum total protein, serum albumin, serum creatinine, and eGFR between 2 groups, which indicate that serum anti-PLA2R antibody is a more robust marker to reflect disease activity than glomerular PLA2R deposition. Moreover, the differences between 2 groups also indicate the patients who had positive glomerular PLA2R deposition and negative serum anti-PLA2R antibody may in the stage 1 or stage 4 of the disease process.

This study was intrinsically limited by its retrospective nature. Furthermore, various therapies given to the patients made it difficult to compare treatment responses. So, the association between serum anti-PLA2R antibody and glomerular PLA2R deposition, and the underlying mechanisms need additional exploration in future studies.

## Acknowledgments

The authors thank Su-Xia Wang for help with pathology consultation, Hong-Yun Yang for help with the use of laboratory information system, and Zi-Long Zhao for assistance in statistics. The authors also thank to all the patients for their participation.

## Supplementary Material

Supplemental Digital Content
