# Supplementary material for: Serum anti-PLA2R antibody and glomerular PLA2R deposition in Chinese patients with membranous nephropathy: A cross-sectional study
Source: Medicine (Baltimore). 2017 Jun 16;96(24):e7218. doi: 10.1097/MD.0000000000007218 (PMC5478356; doi:10.1097/MD.0000000000007218)
Supplement: Supplemental Digital Content [file medi-96-e7218-s001.doc]

**Supplemental Figure Serum anti-PLA2R antibody level of inception group.** Median: 93.00 RU/mL, interquartile range: 23.63-162.38 RU/mL.


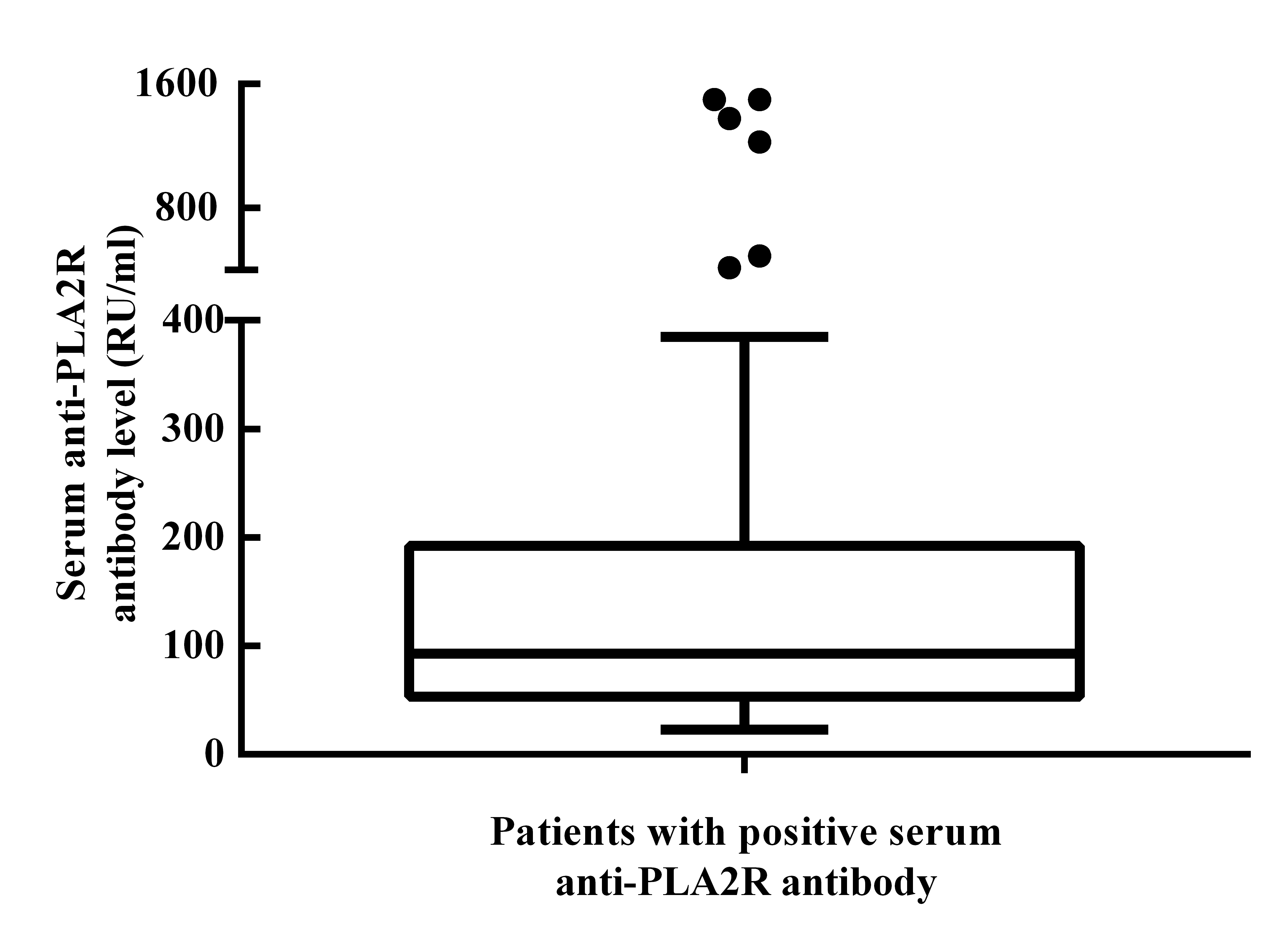


**Supplemental Table. The detail information of the 19 patients who had two kinds** of kidney diseases

| **Pathologic diagnoses** | **N.** |
| --- | --- |
| IgA nephropathy combined with diabetic nephropathy | 5 |
| Focal segmental glomerulosclerosis combined with diabetic nephropathy | 3 |
| IgA nephropathy combined with minimal change disease | 2 |
| ANCA-associated glomerulonephritis combined with diabetic nephropathy | 2 |
| Lupus nephritis combined with thrombotic microangiopathy | 2 |
| Mesangial proliferative glomerulonephritis combined with thin basement membrane nephropathy | 1 |
| Mesangial proliferative glomerulonephritis combined with diabetic nephropathy | 1 |
| Mesangial proliferative glomerulonephritis combined with thrombotic microangiopathy | 1 |
| Crescentic glomerulonephritis combined with IgA nephropathy | 1 |
| Minimal change disease combined with thin basement membrane nephropathy | 1 |
| Total | 19 |
